# An Improved Fusion Paired Group Lasso Structured Sparse Canonical Correlation Analysis Based on Brain Imaging Genetics to Identify Biomarkers of Alzheimer’s Disease

**DOI:** 10.3389/fnagi.2021.817520

**Published:** 2022-01-06

**Authors:** Shuaiqun Wang, Xinqi Wu, Kai Wei, Wei Kong

**Affiliations:** College of Information Engineering, Shanghai Maritime University, Shanghai, China

**Keywords:** sparse canonical correlation analysis (SCCA), GraphNet regularization, Alzheimer’s disease (AD), brain imaging genetics, SNP, gene expression

## Abstract

Brain imaging genetics can demonstrate the complicated relationship between genetic factors and the structure or function of the humankind brain. Therefore, it has become an important research topic and attracted more and more attention from scholars. The structured sparse canonical correlation analysis (SCCA) model has been widely used to identify the association between brain image data and genetic data in imaging genetics. To investigate the intricate genetic basis of cerebrum imaging phenotypes, a great deal of other standard SCCA methods combining different interested structed have now appeared. For example, some models use group lasso penalty, and some use the fused lasso or the graph/network guided fused lasso for feature selection. However, prior knowledge may not be completely available and the group lasso methods have limited capabilities in practical applications. The graph/network guided approaches can use sample correlation to define constraints, thereby overcoming this problem. Unfortunately, this also has certain limitations. The graph/network conducted methods are susceptible to the sign of the sample correlation of the data, which will affect the stability of the model. To improve the efficiency and stability of SCCA, a sparse canonical correlation analysis model with GraphNet regularization (FGLGNSCCA) is proposed in this manuscript. Based on the FGLSCCA model, the GraphNet regularization penalty is imposed in our study and an optimization algorithm is presented to optimize the model. The structural Magnetic Resonance Imaging (sMRI) and gene expression data are used in this study to find the genotype and characteristics of brain regions associated with Alzheimer’s disease (AD). Experiment results shown that the new FGLGNSCCA model proposed in this manuscript is superior or equivalent to traditional methods in both artificially synthesized neuroimaging genetics data or actual neuroimaging genetics data. It can select essential features more powerfully compared with other multivariate methods and identify significant canonical correlation coefficients as well as captures more significant typical weight patterns which demonstrated its excellent ability in finding biologically important imaging genetic relations.

## Introduction

Alzheimer’s disease (AD) is an irreversible long-time neurodegenerative disease and not only brings misfortune to the patient, but also brings a heavy economic and emotional burden to the family (Alzheimer’s Association, 2013). AD is the most common form of dementia and its incidence increases with the aging of the population (Goldberg, 2007). In the past ten years, image genetics has become a crucial research topic in biomedicine and bioinformatics. The reason is that the potential influence of genes on brain structure and function can be found by genetic research. As a powerful tool for data-driven association analysis, statistical learning methods can make full use of the inherent structural information of biomarker data to build models to analyze the correlation between susceptible genes and brain structure or function which can indicate the pathogenesis of brain cognitive behavior or related diseases well. Image genetics can be used to identify the relationship between imaging results and genetic variables (Chen et al., 2013; Hashimoto et al., 2015; Aghakhanyan et al., 2018). Therefore, imaging genetics has become a hot research topic in biomedicine and bioinformatics research.

Correlated canonical analysis (CCA) (Hotelling, 1936) is a classic algorithm and a hot spot in imaging genetics. CCA can be used to mine the correlation between data. However, when using the traditional CCA method, a serious over-fitting phenomenon may appear. For the sake of dealing with this issue, some scholars have introduced sparse canonical correlation analysis (SCCA), which can be used to identify bivariate contacts between a great number of genes and dozens of imaging quantitative traits (QTs). Then, to more effectively distinguish the bivariate correlation about a series of genes with a large number of imaging QTs, some researchers have made different amendments for SCCA. The GraphNet based sparse canonical correlation analysis model (GNSCCA) used graph-constrained resilient network regularization, which not only can find meaningful connections, but also contribute to the smoothness between adjacent coefficients (Du et al., 2015). The an improved GNSCCA method (AGNSCCA) introduced one new penalty to improve SCCA model and developed an effective optimization algorithm to get a better typical correlation coefficient (Du et al., 2016). Sparse canonical correlation analysis based on joint connectivity (JCBSCCA) proposed a connectivity-based penalty measure to incorporate prior biological information and had sound anti-noise performance (Kim et al., 2020). Some scholars have considered that genetic data and imaging features had different group-level structures. Because prior knowledge is not fully available in real life, they improved the lasso penalty combined lasso with graph/network guidance in structured sparse learning. Du et al. (2020) proposed the FGLSCCA (Grosenick et al., 2013) adding two new penalty conditions to the SCCA model, namely, the fusion paired group lasso (FGL) as well as the graph guided paired group lasso (GGL). However, FGLSCCA also has certain shortcomings. The stability and anti-interference of the FGLSCCA algorithm are not good enough, and it cannot incorporate physiological restraints such as connectivity.

In response to the above problems, FGLGNSCCA algorithm is proposed in our present study. First of all, GraphNet regularization (Grosenick et al., 2013) is added to the punitive measure in FGLGNSCCA model. GraphNet regularization is an upgraded version of resilient network regularization and can validly incorporate physiological restraints. Moreover, JCBSCCA has confirmed its stability and noise resistance. To make the model’s results more biological explanatory power, this manuscript applies it as prior knowledge to the model. Secondly, this manuscript derives an efficient iterative optimization algorithm, which proves that the algorithm converges to the optimal local solution. Firstly, we use synthetic data for testing. These experiments illustrate that the algorithm has better noise immunity than other algorithms. When the data set is small, it has more smoothness. Then we use the accurate data set. These results suggest that it has a better canonical correlation coefficient. It is effective to recognize salient features on the actual data set.

## Method

### Sparse Canonical Correlation Analysis

In the formulas, bold lowercase letters represent vectors and bold uppercase letters describe matrices. Expressly, we set **X** ∈ *R^n×p^*, **Y** ∈ *R^n×q^* in this article. **X** has n samples and *p* features, while **Y** has n samples and *q* features. Meanwhile, **X** is the genotype data set as well as **Y** is the image data set. CCA is used to analyze the correlation between two data sets. The purpose of the CCA model is to find the weight vectors **u** and **v** of the features in **X** and **Y** that maximize the relation. The formula is as follows:


(1) $$\begin{array}{c} \underset{u,v}{\text{max}}{\text{u}}^{T}X^{T}Y\text{v}\\ s.t.{\text{u}}^{T}X^{T}Xu={\text{v}}^{T}Y^{T}Yv=1, \end{array}$$

In image genetics, the feature dimensions of data are often much higher than the sample size which lead to over-fitting. Witten et al., proposed sparse SCCA (Parkhomenko et al., 2009; Du et al., 2020) to solve excessive feature dimensionality. The definition is as follows:


(2) $$\begin{array}{c} \underset{u,v}{min}-{\text{u}}^{T}{\text{X}}^{T}\text{Y}v+\lambda_{\text{u}}{||u||}_{1+\lambda_{\text{v}}}^{1}{||v||}_{1}^{1}\\ s.t.{||u||}_{2}^{2}={||v||}_{2}^{2}=1 \end{array}$$

### FGLSCCA Model

Du et al. (2020) imposed two new penalties FGL and GGL on the SCCA model (Grosenick et al., 2013).


(3) $$\begin{array}{c} \underset{u,v}{min}-{\text{u}}^{\text{T}}{\text{X}}^{\text{T}}\text{Y}v+\Omega_{\text{FGL}}\left(u\right)+\Omega_{\text{GGL}}\left(v\right)\\ s.t.{||\mathrm{Xu}||}^{2}\leq 1,{||\mathrm{Yv}||}^{2}\leq 1 \end{array}$$

Among them, the FGL and GGL penalties are defined as:


(4) $$\Omega_{FGL}\left(u\right)=\lambda_{1}\sum_{i=1}^{p-1}\omega_{i,i+1}\sqrt{u_{i}^{2}+u_{i+1}^{2}}$$

(5) $$\Omega_{\text{GGL}}\left(v\right)=\lambda_{2}\sum_{\left(j,k\right)\in E}\omega_{j,k}\sqrt{v_{j}^{2}+v_{k}^{2}}$$

Here, ω_*j*,*k*_ is the weight value of the edge. GGL is an effective technique for estimating the inverse covariance matrix.

### New Connectivity Penalties

This article used a new penalty term based on connectivity, and it was graphed (Grosenick et al., 2013). GraphNet regularization is one restraint by an amended version of the resilient network regularization, which allows the effective integration of physical constraints of connectivity (Grosenick et al., 2013).

First of all, connectivity methods can quantify meaningful neurobiological measurements and are a good source of information (Hagmann et al., 2008). Second, the GraphNet regularization program encourages the similarity of the relevant elements of the canonical vector (Du et al., 2016). The formula is as follows:


(6) $$\begin{array}{c} \text{P}\left(u\right)=\sum_{i,j}{\text{C}}_{u\left(i,j\right)}{\left({\text{u}}_{\text{i}}-{\text{u}}_{\text{j}}\right)}^{2}\\ \text{P}\left(v\right)=\sum_{i,j}{\text{C}}_{v\left(i,j\right)}{\left({\text{v}}_{\text{i}}-{\text{v}}_{\text{j}}\right)}^{2} \end{array}$$

From the literature (Grosenick et al., 2013), the following formula can be obtained:


(7) $$\text{P}\left(u\right)={\text{u}}^{\text{T}}{\text{L}}_{u}\text{u},P\left(v\right)={\text{v}}^{\text{T}}{\text{L}}_{v}\text{v}$$

**L_u_** and **L_v_** mean the Laplacian matrix.

### The Proposed FGLGNSCCA Model

A new structured sparse canonical correlation analysis method (FGLGNSCCA) was proposed in this manuscript. In the presented model, *X* ∈ *R^n×p^* and **Y** ∈ *R^n×q^* represented the gene variable matrix and the brain image variable matrix, respectively. Meanwhile, **u** and **v** represented the characteristic weights or regular loads of ***X*** and ***Y***, respectively.

The model formula is as follows:


(8) $$\begin{array}{c} \underset{u,v}{min}-{\text{u}}^{\text{T}}X^{\text{T}}Yv+\Omega_{\text{FGL}}\left(u\right)+\Omega_{\text{GGL}}\left(v\right)+\frac{\gamma_{1}}{2}\left({||\mathrm{Xu}||}^{2}-1\right)\\ \;+\frac{\gamma_{2}}{2}\left({||Yv||}^{2}-1\right)+\frac{\lambda_{1}}{2}{\text{u}}^{T}L_{u}\text{u}+\frac{\lambda_{2}}{2}{\text{v}}^{T}L_{v}\text{v}\\ \;s.t.{||\mathrm{Xu}||}^{2}\leq 1,{||\mathrm{Yv}||}^{2}\leq 1, \end{array}$$

**FIGURE 1:**
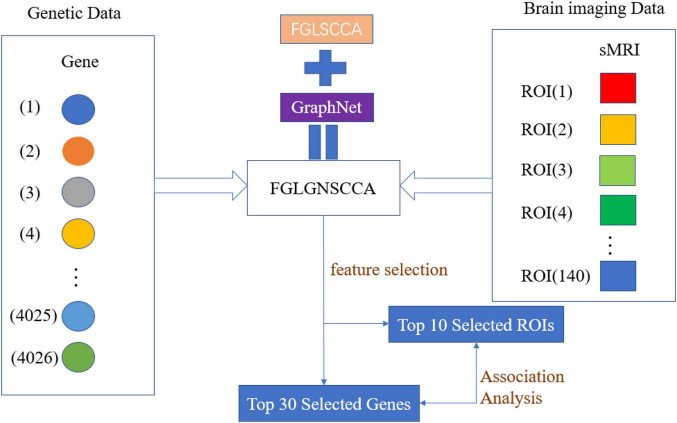
The schematic diagram of the proposed algorithm FGLGNSCCA.

Figure 1 is the schematic diagram of the proposed algorithm FGLSCCA.

### Agency Goals and Optimization Algorithms

If this article directly used the Lagrangian method to find the partial derivatives of **u** and **v** in equation (8), it was quite difficult. Therefore, we used the results of Grosenick et al. (2013), Du et al. (2020) and used the substitution functions $\Omega_{FGL}^{APP}\left(u\right)$ and $\Omega_{GGL}^{APP}\left(v\right)$ derived when processing the data. In addition, set ||**Xu**||^2^ = 1 and ||**Yv**||^2^ = 1, and *L*(**u**, **v**) is as follows:


(9) $$\begin{array}{c} L\left(u,v\right)=-{\text{u}}^{\text{T}}X^{\text{T}}Yv+\Omega_{FGL}^{APP}\left(u\right)+\Omega_{GGL}^{APP}\left(v\right)+\frac{\gamma_{1}}{2}\left({||Xu||}^{2}-1\right)\\ +\frac{\gamma_{2}}{2}\left({||Yv||}^{2}-1\right)+\frac{\lambda_{1}}{2}{\text{u}}^{T}{\text{L}}_{u}\text{u}+\frac{\lambda_{2}}{2}{\text{v}}^{\text{T}}{\text{L}}_{v}\text{v} \end{array}$$

γ_1_, γ_2_, λ_1_, and λ_2_ are artificially set positive tuning parameters, and this Lagrangian function is continuous. Therefore, the vectors **u** and **v** can be differentiated. The partial derivatives of U and V need to be calculated, and then set *L*(**u**, **v**) = 0 to get the extreme value:


(10) $$0=-{\text{X}}^{T}\mathrm{Yv}+\left(\lambda_{1}D_{X}+\gamma_{1}X^{T}\text{X}+\lambda_{1}{\text{L}}_{u}\right)\text{u},$$

(11) $$0=-{\text{Y}}^{T}\text{Xu}+\left[\lambda_{2}{\text{D}}_{Y}+\gamma_{2}{\text{Y}}^{T}\text{Y}+\lambda_{2}{\text{L}}_{v}\right]\text{v},$$

Here, **D_X_** ∈ *R^p×p^* and **D_Y_** ∈ *R^q×q^* are diagonal matrix.


(12) dXii=ωi-1,iui-12+ui2+ωi,i+1ui2+ui+12s.t.ω0,1=ωp,p+1=0,

(13) dYjj=∑m=1,(j,m)∈Eqωj,mvj2+vm2

Here, dXii is the *i*-th element of **D_X_**, and dYjj is the *j*-th element of **D**_*Y*_.

The following formula can be obtained by the formulas (10) and (11):


(14) $$\text{u}=\frac{X^{T}Yv}{\lambda_{1}{\text{D}}_{X}+\gamma_{1}X^{T}X+\lambda_{1}{\text{L}}_{u}},$$

(15) $$\text{v}=\frac{{\text{Y}}^{T}\text{X}u}{\lambda_{2}{\text{D}}_{Y}+\gamma_{2}Y^{T}Y+\lambda_{2}{\text{L}}_{v}},$$

The pseudo code of the model is shown in Table 1.

**TABLE 1:** Pseudo code for FGLGNSCCA.

Algorithm 1: Algorithm for FGLGNSCCA
**Require:** Normalized data **X** ∈ *R^n×p^*,_*Y*∈*R^n×q^*_, _*set*_ parameters λ_1_, λ_2_, γ_1_, γ_2_
**Ensure:** Canonical vectors **u**, **v**
1: Initialize **u** ∈ *R*^_p_×1^, **v** ∈ *R*^*q*×1^
2: **While** not converged **do**
3: Update the diagonal matrix **D_X_**, P(**u**)
4: Fix **v** and solve $\text{u}=\frac{X^{T}\mathrm{Yv}}{\lambda_{1}D_{X}+\gamma_{1}X^{T}X+\lambda_{1}L_{u}}$
5: Scale **u** = **u**./*sqrt*(**u^T^X^T^Xu**)
6: Update the diagonal matrix ***D_Y_***, P(**v**)
7: Fix **u** and solve $\text{v}=\frac{Y^{T}\mathrm{Xu}}{\lambda_{2}D_{Y}+\gamma_{2}Y^{T}Y+\lambda_{2}L_{v}}$
8: Scale **v** = **v**./*sqrt*(**v**^T^***Y***^*T*^***Y*v**)
9: End while

## Results

### Simulation Data Experiment

**FIGURE 2:**
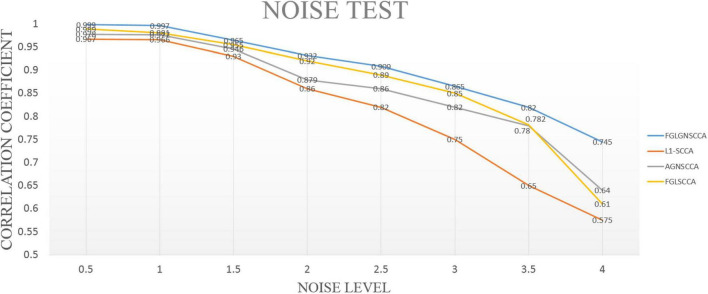
The test results of different models under different noise levels. The horizontal axis is the noise level, and the vertical axis is the typical correlation coefficient.

In this part, simulated data has been used for experiments. Therefore, the accuracy of the proposed algorithm for detecting highly correlated biomarkers can be more intuitively estimated. First, we simulated the generation of two loading vectors as ground truth to simulate gene and image features. The number of the samples was set up *n*. In the data (gene data and image data), the gene data had p = 800 feature dimensions, and the image data had *q* = 100 dimensions. Secondly, this manuscript generated a latent variable ε*N*(0, δ^2^) to express the correlation between genetic data and images (Lin et al., 2014). Finally, this manuscript imposed different noise levels on the generated data matrix to evaluate the anti-noise performance of the model. We compared with the proposed model with FGLSCCA, L1-SCCA, AGNSCCA as shown in Figure 2 which shown the influence of different noise levels on the sample correlation results under 100 times of fivefold cross-validation.

In Figure 2, it can be seen that as the noise level continues to increase, the calculated typical correlation coefficients of each model are decreasing, and the stability of the correlation results also decreases to varying degrees. Under the low-level noise, the difference in the typical correlation coefficients of different models is slight, but the proposed algorithm FGLGNSCCA still has a weak advantage. Under the high-level noise, the new model presented has higher correlation typical coefficients. Therefore, our model is better than other three models. In general, under the same conditions, the model proposed in this manuscript has better anti-noise performance and sample correlation, which is more conducive to the analysis of data correlation results and the discovery of the pathogenic mechanism of AD’s related biomarkers.

### Subject Data and Preprocessing

The genetic data and imaging phenotype data used in this article are all from the Alzheimer’s Disease Neuroimaging Project (ADNI) database^1^. The main contribution of ADNI is the development of clinical, imaging, genetic and biomarkers for early detection and tracking of AD.

Consistent with the previous preprocessing method, this article downloaded the data of 386 non-Hispanic white subjects in ADNI1, including imaging and genotyping data (Wei et al., 2021). First, for raw structure magnetic resonance imaging (sMRI), DiffusionKit (Gorski et al., 2007) is used to perform head movement correction on sMRI. Secondly, using the SPM software package (Saykin et al., 2010). CA T toolkit to achieve sMRI segmentation, the image phenotype feature comprises 140 regions of interest (ROI).

This article uses PLINK (Jung and Hu, 2015) to preprocess the genotype data and screen it according to the following criteria: *HWEp* < 10^−6^, extract genes with variance more significant than 0.5. In the end, 4,026 genes were obtained in this article. The characteristics of the subjects are counted in Table 2.

**TABLE 2:** Characteristics of the subjects.

Groups	AD	EMCI	LMCI	HC
Number	25	186	62	113
Gender (M/F)	10/15	101/85	32/30	58/55
Age (mean ± SD)	75.99 ± 10.22	71.56 ± 7.51	72.91 ± 6.82	75.06 ± 5.68

*EMCI stands for Early Mild Cognitive Impairment, LMCI stands for Late mild cognitive impairment, HC stands for Healthy Contro.*

### Experimental Setup and Parameter Selection

In this part, this article will use the algorithm to experiment on accurate data, and finally select the appropriate parameters. In the FGLGNSCCA model, there are four parameters (λ_1_, λ_2_, γ_1_, γ_2_) that need to be set manually. In this study, the values of λ_1_ and λ_2_ will be fixed, and the values of γ_1_ and γ_2_ will be constantly changed for experimentation. When a certain set of values makes the experiment get the largest canonical correlation coefficient, then a set of parameters needed in this research is obtained.

Because of the limited number of samples collected in this article, this article finally chose fivefold cross-validation (Wang et al., 2010). After a complete fivefold cross-validation, this study obtained five typical correlation coefficients (CC).

**FIGURE 3:**
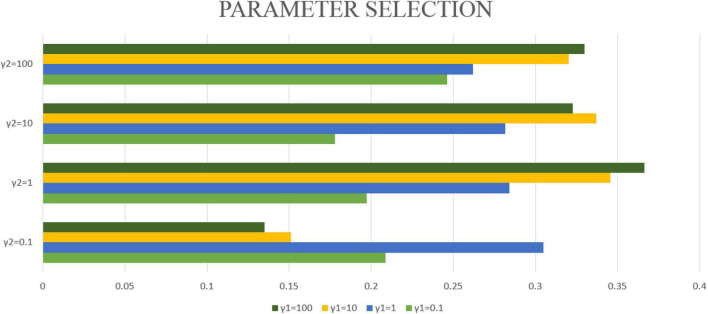
The bar graphs of different colors represent different γ_1_, the vertical axis coordinate represents different γ_2_, and the horizontal axis coordinate represents the typical correlation coefficient under different γ_1_ and γ_2_ conditions.

In this article, λ_1_ = λ_2_ = 1 will be fixed. This article applies the proposed algorithm to image data and gene expression data. The goal of this article is to obtain the most significant canonical correlation coefficient (CC) between gene and image data. Therefore, when the CC is the largest, the parameter results required in this article can be obtained. Then by repeating the experiment 50 times, the average CC and standard deviation are calculated, which are used as the experimental results of this article. However, the blind grid search of parameters is very time-consuming. Therefore, this article matches the values of γ_1_ and γ_2_ one by one from (0.1, 1, 10, 100). After testing with different parameters, γ_1_ = 100 and γ_2_ = 1 are selected in this article. Finally, the maximum correlation coefficient of the model in this manuscript is CC = 0.3665 ± 0.0126. The correlation coefficients obtained by different parameters are shown in Figure 3.

### Experimental Results of Real Data

**FIGURE 4:**
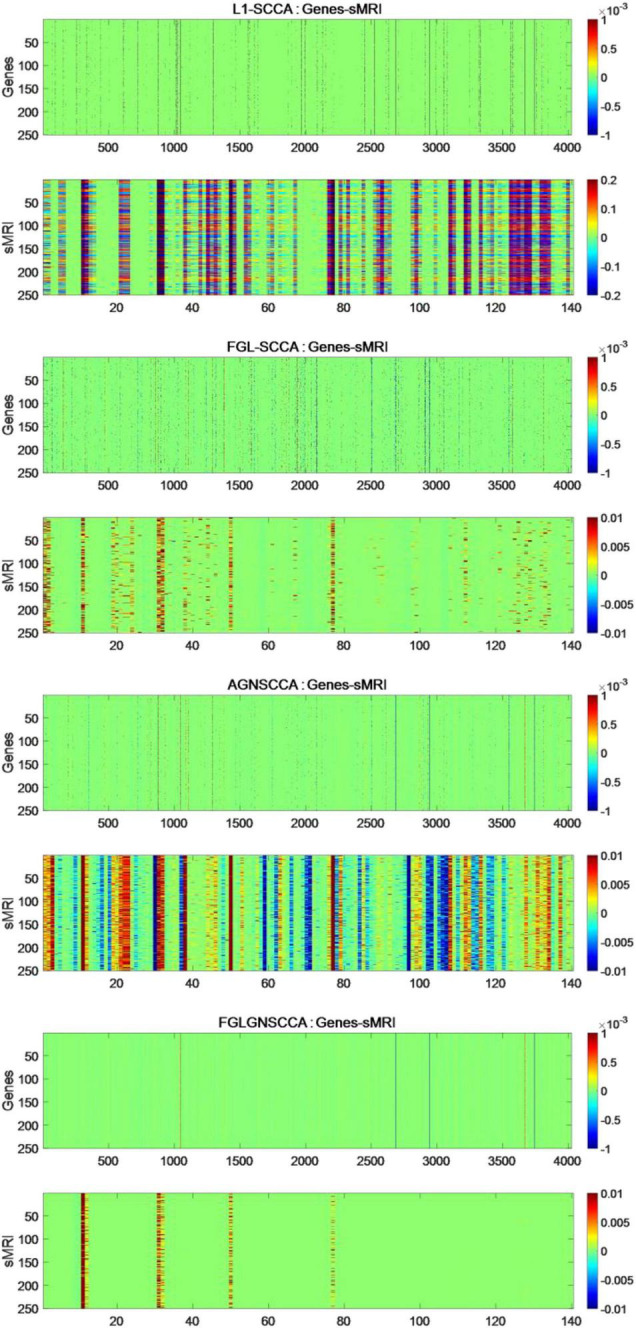
The heat maps obtained by 250 runs of different models. The upper figure in each part is the canonical weight of the genes which is u and the following figure is the image canonical weight which is v. The dimension of standard gene weight is *R*^250×4026^ (each row represents the number of the algorithm runs, and each column represents a feature). The size of the standard sMRI weight is *R*^250×140^.

The fresh model proposed in this study does not use the common generalized fusion lasso, but uses the penalty term using FGL, GGL, and GraphNet normalized form. This study selected 386 sample data, including genetic data and image data. This manuscript compares the FGLGNSCCA model with other models, and finally can confirm whether the algorithm in this manuscript has better performance. To ensure the reliability of the experimental results, this manuscript uses FGLGNSCCA and the other three models to conduct 50 times fivefold cross-validation training, respectively. Each time, a load vector is generated and stored in the matrix. In the end, this research will get a 250 × 4026 matrix and a 250 × 140 matrix. For the above research results, respectively, as shown in Figure 4.

It can be seen from Figure 4 that the L1-SCCA model cannot accurately identify the brain regions and genes from a large amount of data. Although the FGLSCCA and AGNSCCA models can identify a certain number of brain regions and genes, some of their features show disorder and do not have excellent stability. The models of L1-SCCA,FGLSCCA, and AGNSCCA extract too many feature genes and brain regions, which may not be used as effective biomarkers related to AD. First of all, the heat map of the FGLGNSCCA algorithm in Figure 4 clearly displayed the significant genes and brain regions, which is helpful for accurate positioning. Secondly, fewer distinctive features eliminate some interferences, and may help drug research for the treatment of AD. In general, the method in this manuscript was more conducive to discovering relevant biomarkers for the pathogenesis of AD by analyzing the correlation and biological significance between gene expression data and sMRI.

In addition, the TOP10 brain regions identified by the proposed model has been shown and the absolute values of the average weight of 50 × 5 times are listed in Table 3. Due to the high dimensionality of the genes, this article separately displayed the TOP30 genes and average weights identified by the new model proposed in this article in Table 4. At the same time, this article also gave the typical correlation coefficients (Mean ± SD) between gene and sMRI of different models. Through 50 times fivefold cross-validation, the comparison results of canonical correlation coefficients are shown in Table 5.

**TABLE 3:** TOP10 Brain ROI.

ROI	Weight
lCau rThaPro rAngGy lVenVen lMedFroCbr rCau rSupMarGy rPosIns rCbeLoCbe6-7 rPoCGy	3.39E-02 1.24E-02 5.22E-03 2.63E-03 2.31E-03 2.14E-03 9.65E-05 7.22E-05 6.15E-05 5.37E-05

**TABLE 4:** TOP30 gene genetic feature weight.

Gene	Weight
PRKY	9.79E-03
RPS4Y1	8.62E-03
PRKX| | PRXY	8.37E-03
RPS4Y2	6.81E-03
KDM5D	5.26E-03
EIF1AY	4.83E-03
TXLNG2P	4.51E-03
DDX3Y	3.54E-03
UTY	3.19E-03
XIST	2.76E-03
KDM6A	1.03E-03
EIF1AX	7.13E-04
TXLNG	6.23E-04
TTTY10	3.08E-04
DDX58	1.23E-04
USP9Y	1.13E-05
ZFX	9.10E-06
DDX3X	8.64E-06
PPAPDC1B	8.51E-06
POU2AF1	6.65E-06
ZFY	6.55E-06
DDX60	6.01E-06
FCRL1	3.60E-06
NT5E	3.21E-06
PTPRK	2.37E-06
CXCR5	2.09E-06
E2F5	1.23E-06
AFF3	1.15E-06
CXCL5	1.08E-06
FCRL2	9.98E-07

**TABLE 5:** Canonical correlation coefficients of different models.

Model	CC (Mean ± SD)
FGLGNSCCA	0.3665 ± 0.0126
FGLSCCA	0.2891 ± 0.0296
AGNSCCA	0.3056 ± 0.0362
L1-SCCA	0.3102 ± 0.0281

**FIGURE 5:**
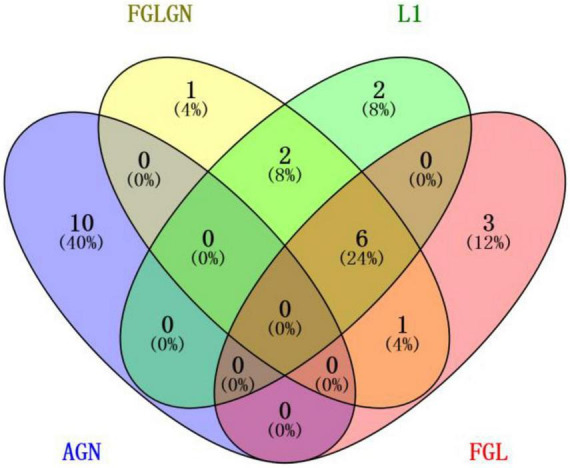
The venn diagram of the brain area.

**FIGURE 6:**
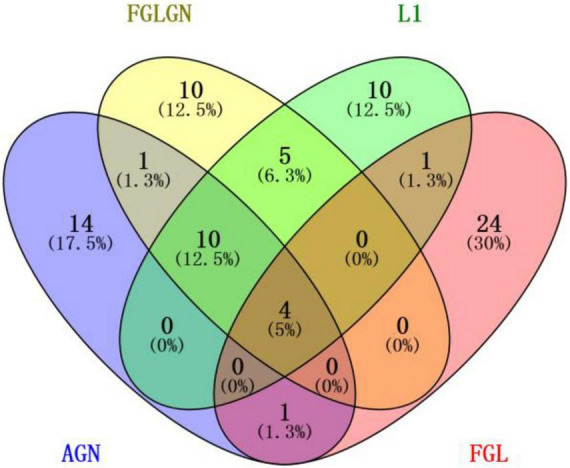
The venn diagram of the gene.

This research, respectively, counted the TOP30 genes and the TOP10 brain regions obtained by the four algorithms, and respectively, drew the gene venn diagram and the brain region venn diagram as shown in Figures 5, 6 (Jia et al., 2021). It can be seen from Figure 6 that the FGLGNSCCA algorithm has obtained ten genes that are not duplicated with other algorithms. The genes, E2F5 and PTPRK, have been confirmed to be related to AD. In the venn diagram of the brain area, the TOP10 brain areas selected by AGNSCCA are not repeated with other algorithms, indicating that the effect of AGNSCCA is not good. FGLGNSCCA, L1-SCCA, and FGLSCCA obtained a total of six identical brain regions, some of which proved to be related to AD, while FGLGNSCCA alone has a brain region named Right Caudate (rCau), which may be a biomarker of AD. With FGLGNSCCA algorithm, more AD-related biomarkers have been found. Therefore, the algorithm proposed in this manuscript is more superior.

## Discussion

In the research of this article, this article used data from 386 samples, including genetic data and image data. When comparing with different models, the new models presented in this article all show better performance. First of all, the new model proposed in this article can display several brain areas more prominently. In contrast, the display of other models is more confusing and cannot effectively identify the prominent brain areas. Secondly, the new model proposed in this article can also identify significant genes and the correlation between image and genetic data, which is incomparable to the other three models.

### Prediction of Region of Interest

**FIGURE 7:**
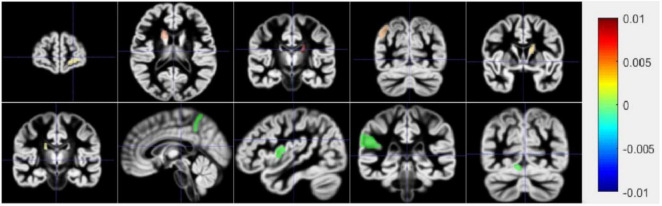
The first row is 1–5 brain areas, and the second row is 6–10 brain areas.

Figure 7 shows a schematic diagram of the first ten brain regions. The color in Figure 7 represents the typical weight of the TOP10 brain regions, which is **v**. The value indicated by the color has been shown on the right side of the picture. The new model proposed in this manuscript identifies the first ten brain regions, among which Left Supramarginal Gyrus (lSupMarGy) (Penniello et al., 1995), Right Thalamus Proper (rThaPro) (de Jong et al., 2008), Left Caudate (lCau) (Baik et al., 2021), and Left Medial Frontal Cerebrum (lMedFroCbr) (Johannsen et al., 1999) are associated with AD. And Left Caudate has the most remarkable correlation in the recognition results of this manuscript, so it further proves the reliability and authenticity of the algorithm in this manuscript, which is due to the excellent performance of the algorithm in this manuscript. Although the other three algorithms can also identify a certain number of brain regions to a certain extent, the algorithm in this article has significant differences. It can identify brain regions that are significantly related to AD. In addition, the Right Angular Gyrus (rAngGy) and self-awareness are functionally associated with the physical disconnection (de Boer et al., 2020), which may be related to the loss of self-awareness in patients with advanced AD. Moreover, Right Angular Gyrus plays an essential role in language function (Rosselli et al., 2015), which may be related to a series of symptoms such as aphasia in AD patients. Right Angular Gyrus, which has a high correlation, has not yet been confirmed to be highly correlated with AD. This may be the next direction for clinical research.

### Over-Representation Analysis

#### Gene Ontology Enrichment Analysis

**FIGURE 8:**
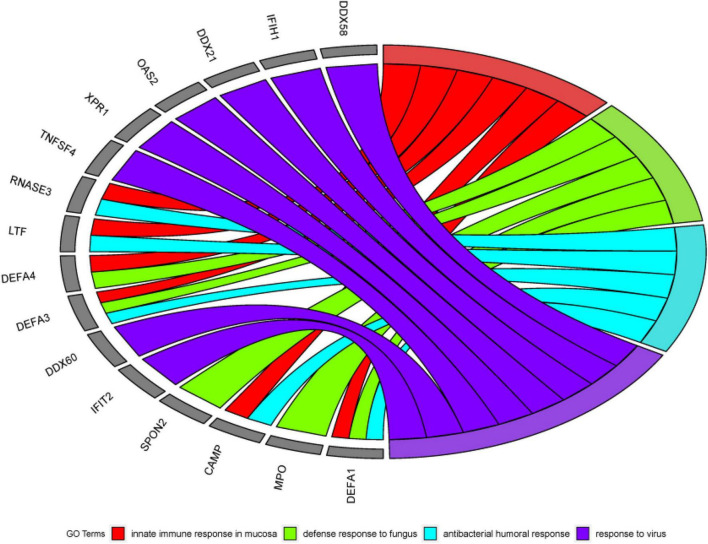
Chord diagram obtained through GO analysis in the experiment. Among the first 500 genes entered, 16 genes were significantly enriched in 4 GO term.

DAVID is a robust database. It has two absolute advantages. First, there are many identifiers. Second, there are many types of background species. It has data on a small number of research objects, and its operation is convenient. Since 2003 Since its inception, it has always had a good reputation. Therefore, this article chooses the DAVID database for data analysis. First, this article uses DAVID Bioinformatics Resources 6.8^2^ to perform gene ontology (GO) enrichment analysis on the first 500 genes identified by the algorithm in this article (Ding and Zhang, 2017). In the results of GO enrichment analysis, this article finally selected the first four more significant terms, as shown in Table 6. A total of 16 different genes are enriched in these four terms. From this result, it can be concluded that these 16 genes are all involved in biological processes (BP), and the detailed GO enrichment analysis is shown in the GO string diagram Figure 8.

**TABLE 6:** Four sets of significant terms obtained by GO analysis.

Category	ID	Term	FDR
BP	GO:0002227	innate immune response in mucosa	6.24E-02
BP	GO:0050832	defense response to fungus	6.24E-02
BP	GO:0019731	antibacterial humoral response	7.99E-02
BP	GO:0009615	response to virus	7.99E-02

Alzheimer’s disease not only damages the human brain, but also can cause damage to other human organs. The early stage of AD is not fatal, but in the middle and late stages, AD will bring various complications (heart disease, thromboembolism, stroke, and renal failure, etc.), which will bring death threats to the patient. From this, we know that AD is not a simple neurological disease, but a comprehensive disease. Mental functions such as early and mid-term characteristic memory of AD patients are weakened. Still the late symptoms of AD are aphasia, a decline in physical fitness and loss of bodily control. It can be seen that AD is a chronic disease with multiple genes working together, and its pathogenesis includes a large number of biological processes. Because AD’s toxic proteins can erode brain cells, the innate immune response of the brain mucosa is a critical protective mechanism. It can also be seen in the analysis results, innate immune response in the mucosa is one of the most effective terms. It has been confirmed in the literature (Stylianaki et al., 2019) that when the antibacterial response of neutrophils outside the patient’s body is damaged, the probability of getting sepsis will increase, and sepsis is one of the complications of AD. This can also be reflected in the analysis of this article. That is, the antibacterial humoral response is one of the first four significant terms. The above reveals the link between some diseases and AD.

#### Kyoto Encyclopedia of Genes and Genomes Pathway Analysis

**FIGURE 9:**
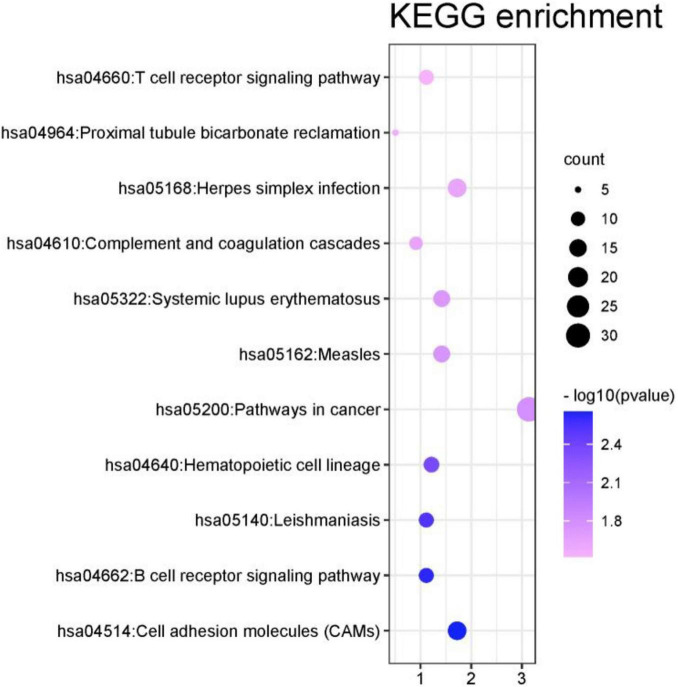
KEGG enrichment analysis results of the first 1000 genes.

In this part, this article also used the DAVID database to perform the Kyoto Encyclopedia of Genes and Genomes (KEGG) pathway analysis on the first 1000 genes identified by the algorithm (Kanehisa et al., 2017). The DAVID database identified 987 genes. Other genes did not match. It may be because the database has not been updated in time or the gene names are outdated. Among all the genes compared to the database, a total of 351 genes were enriched in the KEGG signal path, accounting for about 35.6%. To observe the significance of the input gene enrichment in the pathway, after artificially setting *P*-value < 0.05, 11 signal pathways were screened, as shown in Figure 9.

**FIGURE 10:**
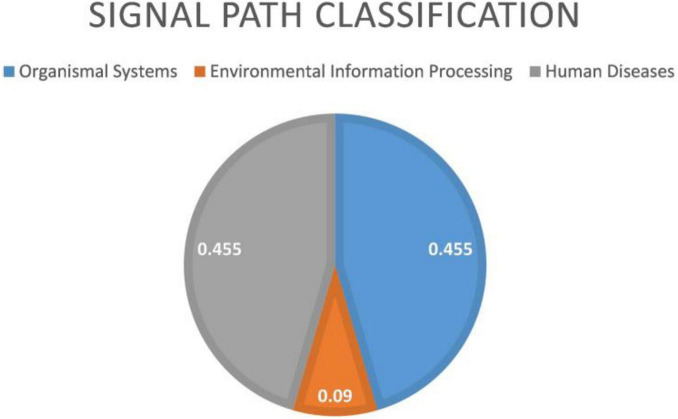
Environmental information processing includes 1 signal path. Organismal systems includes five signal paths; human diseases includes five signal paths.

From Figure 9 that the cell adhesion molecules (CAMS) signal pathway enrichment analysis is more significant than other pathways. In the literature (Leshchyns’ka and Sytnyk, 2016), it is shown that the loss of synapses between brain neurons is inevitable with Alzheimer’s disease (AD). The article describes in detail that changes in synaptic adhesion play a vital role in the destruction of neuronal networks in AD. From Figure 10 that these 11 signal pathways can be divided into three major categories, namely environmental information processing, Organismal Systems, and Human Diseases. The signal pathways we have identified are highly related to organism systems and human diseases.

The above analysis proves that the new algorithm proposed in this manuscript has identified the signal pathways related to AD, proving that FGLGNSCCA has powerful performance.

### Refinement Analysis

Among the TOP30 genes identified by FGLGNSCCA, genes such as ZFX (Soleimani et al., 2020), XIST (Wang et al., 2018), E2F5 (Johanson et al., 2008), KDM6A (Davis et al., 2020), TXLNG (Hotokezaka et al., 2015), and PTPRK (Chen et al., 2018) have been confirmed to play an eventful role in the AD process or participation in related biological processes. The RPS4Y1 gene is associated with Parkinson’s disease (Sun et al., 2014). The literature (Yue et al., 2020) deemed that XIST may become a new underlying aim for the remedy of AD. At the same time, the literature (Chanda and Mukhopadhyay, 2020) also discussed the possibility of XIST-mediated therapeutic intervention and the relationship between XIC and women’s preference for AD. The PTPRK gene is associated with an increased risk of neuropsychiatric diseases and cancer, and the literature (Sun et al., 2014) provided evidence that the PTPRK gene is associated with the risk of AD. The relationship between other genes and AD needs to be studied in the future.

**FIGURE 11:**
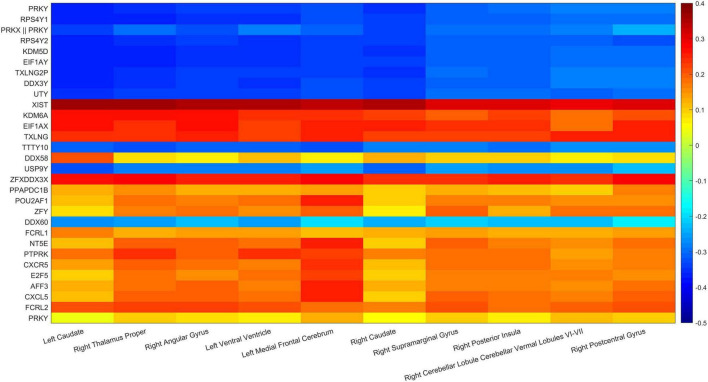
Gene-ROI pairwise correlation.

In addition, the paired correlation heat maps of TOP30 genes and TOP10 brain regions are shown in Figure 11 in this article which in Tables 3, 4. The *Y*-axis direction is the typical weights of genes arranged from small to large, and the *X*-axis is the typical weights of brain regions from high to low. As expected in this article, all Gene-ROI pairs have a strong correlation. And it can be observed in this article that the first nine genes (PRKY, RPS4Y1, PRKX | | PRKY, RPS4Y2, KDM5D, EIF1AY, TXLNG2P, DDX3Y, and UTY) are negatively correlated with all brain regions. And it can be found that the effects of the same genetic variable on different brain regions show the same positive or negative relationship as a whole.

This article used the z-test to analyze the pairwise correlation of the Gene-ROI we got. The number of Gene-ROI pairwise correlation coefficient is 300, z-test is selected in this article. Next, this article selected the TOP10 data with *p*-value less than 0.01 which are shown in Table 7. It can be seen from the Table 7 that the XIST gene is extremely related to five brain regions, and the XIST gene has been confirmed to be related to AD. Caudate brain region is extremely related to six genes, and Caudate has also been shown to be related to AD. Therefore, this article believes that XIST gene and Caudate brain region are likely to be biomarkers of AD.

**TABLE 7:** The TOP10 pairs with *p* <0.01.

Gene-ROI	*P*-value
XIST-Left Caudate	0.00291
XIST-Right Thalamus Proper	0.00366
XIST-Left Ventral Ventricle	0.00441
XIST-Right Angular Gyrus	0.00470
KDM5D-Left Caudate	0.00534
XIST-Right Caudate	0.00546
RPS4Y2-Left Caudate	0.00577
PRKX | | PRKY-Left Caudate	0.00597
EIF1AY-Left Caudate	0.00604
TXLNG2P-Left Caudate	0.00609

Of course, the algorithm proposed in this article also has certain shortcomings. First of all, the collected samples limited the performance of the model. Due to the small number of samples, various penalty items may cause over-fitting problems. At the same time, we have not collected more image data to get a closer image genetic association in addition to sMRI.

## Conclusion

This article dedicates identifying biomarkers related to AD through image genetics. Once they are clinically verified, they can better predict the possibility of a person becoming an AD patient and guide clinical decision-making. In this study, this manuscript adds GraphNet regularization based on FGLSCCA. GraphNet regularization is a constraint by a modified version of the resilient network regularization, which allows the physical limitations of connectivity to be effectively integrated. First of all, in the research using artificially synthesized highly correlated data sets for testing, these findings indicate that the algorithm in this manuscript has better anti-noise capability than the three methods (L1-SCCA, FGLSCCA, and AGNSCCA). Secondly, on the actual ADNI data set, we used the data set of 386 non-Hispanic white subjects. After the FGLGNSCCA model was run through 50-fold cross-validation, it obtains a higher canonical correlation coefficient of gene-ROI than other models, and more significant biomarkers have been identified. Again, this article uses the David database in the biological analysis. In the GO and KEGG enrichment analysis, this study found that 16 genes are present in 4 significant GO Term, and 351 genes are present in 11 signal pathways. These intuitive biological analyses can make it easier for us to interpret AD pathology-related problems. Finally, by displaying the pairwise correlation heat map of genetic variables and image variables, this article shows that the effects of the same gene on different brain regions are all related in the same direction as a whole. And we found a combination of ROI and gene, and this combination may be contacted to AD. It further shows the close relationship between genetic variables and brain regions. In the future, we will undertake to add other data together for research, hoping to more effectively explore the biological relationship between genetic data and imaging data.

Most people only think that AD is a chronic neurological disease, which only has the characteristics of dementia, memory loss, and other non-lethal features. But in fact, AD is a fatal chronic neurological disease. The early stage of AD is just some trivial things such as memory decline, and these things will naturally occur with age, and people will naturally not pay more attention. But in most cases, when a person is diagnosed as an AD patient, his condition has reached the middle or late stage, and at this time, the doctor is unable to recover. Therefore, it is hoped that the new algorithm proposed in this article can effectively and earlier identify patients with early AD or ordinary people who may become AD patients.

## Data Availability Statement

The original contributions presented in the study are included in the article/supplementary material, further inquiries can be directed to the corresponding author.

## Author Contributions

SW, XW, and WK: research conception and design. XW and KW: data collection, analysis, and interpretation. XW and SW: statistical analysis and manuscript drafting. SW, XW, KW, and WK: reviewing important academic content. All authors contributed to the article.

## Conflict of Interest

The authors declare that the research was conducted in the absence of any commercial or financial relationships that could be construed as a potential conflict of interest.

## Publisher’s Note

All claims expressed in this article are solely those of the authors and do not necessarily represent those of their affiliated organizations, or those of the publisher, the editors and the reviewers. Any product that may be evaluated in this article, or claim that may be made by its manufacturer, is not guaranteed or endorsed by the publisher.

## Footnotes

1
http://ADNI.loni.usc.edu/

2
https://david.ncifcrf.gov/
